# The Essential Thioredoxin Reductase of the Human Pathogenic Mold *Aspergillus fumigatus* Is a Promising Antifungal Target

**DOI:** 10.3389/fmicb.2020.01383

**Published:** 2020-06-25

**Authors:** Jasmin Binder, Yana Shadkchan, Nir Osherov, Sven Krappmann

**Affiliations:** ^1^Mikrobiologisches Institut – Klinische Mikrobiologie, Immunologie und Hygiene, Universitätsklinikum Erlangen and Friedrich-Alexander-Universität Erlangen-Nürnberg, Erlangen, Germany; ^2^Aspergillus and Antifungal Research Laboratory, Department of Clinical Microbiology and Immunology, Sackler School of Medicine, Tel Aviv University, Tel Aviv-Yafo, Israel; ^3^Medical Immunology Campus Erlangen, Friedrich-Alexander-Universität Erlangen-Nürnberg, Erlangen, Germany; ^4^Erlangen Center of Infection Research, Friedrich-Alexander-Universität Erlangen-Nürnberg, Erlangen, Germany

**Keywords:** aspergillosis, essentiality, conditional promoter replacement, thioredoxin, TetOFF

## Abstract

The identification of cellular targets for antifungal compounds is a cornerstone for the development of novel antimycotics, for which a significant need exists due to increasing numbers of susceptible patients, emerging pathogens, and evolving resistance. For the human pathogenic mold *Aspergillus fumigatus*, the causative agent of the opportunistic disease aspergillosis, only a limited number of established targets and corresponding drugs are available. Among several targets that were postulated from a variety of experimental approaches, the conserved thioredoxin reductase (TrxR) activity encoded by the *trxR* gene was assessed in this study. Its essentiality could be confirmed following a conditional TetOFF promoter replacement strategy. Relevance of the *trxR* gene product for oxidative stress resistance was revealed and, most importantly, its requirement for full virulence of *A. fumigatu*s in two different models of infection resembling invasive aspergillosis. Our findings complement the idea of targeting the reductase component of the fungal thioredoxin system for antifungal therapy.

## Introduction

Fungi as infectious agents of man pose a constant threat to a specific cohort of individuals, mainly immunosuppressed patients but also people living in areas being endemic for primary fungal pathogens (Brown et al., 2012; Köhler et al., 2017). With respect to pathogenicity and the evolution of virulence determinants in the fungal kingdom, several scenarios have been conceived, among them the idea that the primary environmental niche may serve as a training ground and virulence school, resulting in the emergence of specific traits that support fitness, resistance, or invasion during infection (Brunke et al., 2016). This is of special relevance when considering opportunistic fungal pathogens that usually inhabit specific ecological sites in the wild to become only pathogenic when encountered by a susceptible, immunocompromised host. In this context, enhanced resistance to various stressors has emerged as a key feature of pathogenic fungi (Hartmann et al., 2011), like against adverse environmental conditions that are mirrored to some extent during the infectious process (Tekaia and Latgé, 2005). Febrile conditions mounted during infections may be countered by fungal thermotolerance, or limited supply of macro- or microelements conferring nutritional immunity by metabolic versatility (Casadevall et al., 2003). Among the most prominent mechanisms of host antifungal resistance raised by immune effector cells are chemically reactive molecules, such as reactive oxygen or nitrogen species, ROS and RNS, respectively. Accordingly, resistance against oxidative stress has to be considered as a paramount feature of fungal pathogens, which is supported by numerous studies demonstrating that corresponding detoxification systems contribute to the virulence potential of a given fungal pathogen (Brown et al., 2007; Leal et al., 2012; Dantas Ada et al., 2015; Brown and Goldman, 2016; Hillmann et al., 2016; Shlezinger et al., 2017).

Among the evolved cellular systems that confer oxidative stress resistance in fungi, sulfur-based ones are of special interest due to their unique characteristics and redox properties. Microbial enzymes commonly scavenge reactive oxygen species by transforming them into less active, non-toxic ones following various reactive paths. The thioredoxin/thioredoxin reductase (Trx/TrxR) pair has gained increasing attention in the past to serve as a thiol-based paradigm system that affects various cellular functions and processes beyond antioxidant defense, including redox homeostasis, regulation of gene expression, or nucleic acid synthesis (Arner and Holmgren, 2000; Mustacich and Powis, 2000; Nordberg and Arner, 2001). Trxs are relatively small oxidoreductases of 12 to 13 kDa and are ubiquitously found in all kingdoms of life. Based on a reactive cysteine pair in the active site they are able to switch between an oxidized disulfide and a reduced dithiol state. TrxRs catalyze the reduction of oxidized thioredoxins, using NADPH as electron donor and FAD as cofactor, which then further reduce disulfides in corresponding target proteins. Generally, TrxRs are homodimeric flavoproteins belonging to the pyridine nucleotide-disulfide oxidoreductase family, which also comprises glutathionreductases and others. They are classified as high or low molecular weight isoforms, with the former ones having evolved in higher eukaryotes and the latter ones in prokaryotes, archea, plants and fungi. Based on significant structural differences between these isoforms, TrxRs have gained attention as cellular targets for antimicrobial compounds that would interfere with low molecular weight class members. The nitroimidazole drug metronidazole, for instance, is reduced by the action of a TrxR in *Entamoeba histolytica* that, in turn, forms adducts with its metabolites (Leitsch et al., 2007). To date, several inhibitors of TrxRs have been identified that may serve as anticancer agents or antibacterial drugs (Saccoccia et al., 2014).

The thioredoxin system of the model fungus *Aspergillus nidulans* has been characterized previously to reveal that growth deficiency of a corresponding Trx deletion mutant could be rescued by glutathione supplementation (Thön et al., 2007). For the yeast *Saccharomyces cerevisiae* it was demonstrated that its TrxR is required for 3′-phosphoadenylsulfate (PAPS) reductase activity and therefore sulfate assimilation (Schwenn et al., 1988; Thomas et al., 1990, which implies relevance of the Trx/TrxR system for fungal sulfur metabolism.

Recently it has been demonstrated that the established drug ebselen that targets TrxR enzymes of bacteria, fungi, or mammals (Azad and Tomar, 2014), strongly inhibits the *A. fumigatus* TrxR (Marshall et al., 2019). This is of special relevance as validated cellular targets and corresponding antifungal compounds are limited, raising severe concerns about future development of antimycotic therapy (Denning and Bromley, 2015). *A. fumigatus* is the main causative agent of aspergillosis, an often severe complication of human health that manifests in a variety of clinical scenarios (Latgé and Chamilos, 2019). Among these, invasive aspergillosis that can affect severely immunocompromised patients is associated with significantly high mortality rates and is therefore regarded as a life-threating opportunistic infection. Based on an *in silico* metabolic network modeling approach, putative targets of antifungal therapy had been identified taking into account conditional expression data together with enzyme structures and interconnectedness with pathogen-specific relations of orthology (Kaltdorf et al., 2016). Among the identified 64 gene targets, several were scored as highly ranked and promising candidates for drug targets. In this respect, the *A. fumigatus* Trx/TrxR system became of interest due to limited structural similarity with the human orthologous system and its high expression *in vivo* during infection in a murine model of invasive pulmonary aspergillosis (McDonagh et al., 2008).

In this present study we demonstrate essentiality of the TrxR-encoding *trxR* gene for *A. fumigatus* and describe its characterization by a conditional promoter replacement strategy with respect to oxidative stress resistance, *trxR* transcription, and virulence, making a case for the sulfur-based redox-active Trx/TrxR system to be recognized as potential antifungal target.

## Results

### Thioredoxin Reductase of *Aspergillus fumigatus* Is Encoded by the Essential trxR Gene

Making use of the *Aspergillus* genome database AspGD^1^ to query the *A. fumigatus* sequence, a clear and single ortholog of fungal TrxR-encoding genes could be identified. The encoding Afu4g12990 locus had been annotated as *trr1* based on the similarity of its gene product to the yeast TrxR. In line with the genetic nomenclature and resembling the designation established in *A. nidulans* (Thön et al., 2007), the gene alias used in this study is *trxR*. This gene spans a coding sequence of 1272 base pairs to comprise three exons that are interrupted by two introns. The deduced protein is 392 amino acids in length with a calculated molecular mass of 42.2 kDa. Submitting its primary sequence to BLAST revealed limited similarity of the *A. fumigatus trxR* gene product to the human orthologous TrxR or its isoforms, with a maximum identity of 28% (data not shown).

Based on former priorization studies complemented by preliminary data from an essential gene identification survey, we made further efforts to characterize the TrxR-encoding gene in *A. fumigatus* by a gene targeting approach. First and to confirm its essentiality, a gene replacement cassette was constructed to delete the *trxR* coding sequence by fusing 5′ and 3′ flanking regions to a resistance-conferring genetic marker. From the resulting plasmid pSK654, a DNA fragment could be excised and transformed into the recipient strain AfS35. From the resulting pool of primary transformants that had been selected in the presence of hygromycin B, conidia were transferred onto medium without selection pressure and scored for growth. For the few picks that were able to germinate and form a mycelium on minimal medium lacking the antifungal, diagnostic PCR confirmed the maintenance of the targeted gene in the transformants’ genome and therefore ectopic integration of the genetic marker cassette into the recipient’s genome (data not shown). Assuming that integration of the marker in an essential gene locus would cause inviability of the resulting conidia, accompanied by growth of only heterokaryotic mycelia, essentiality of the *trxR* gene for *A. fumigatus* could be validated by this heterokaryon rescue approach (Osmani et al., 2006).

**FIGURE 1:**
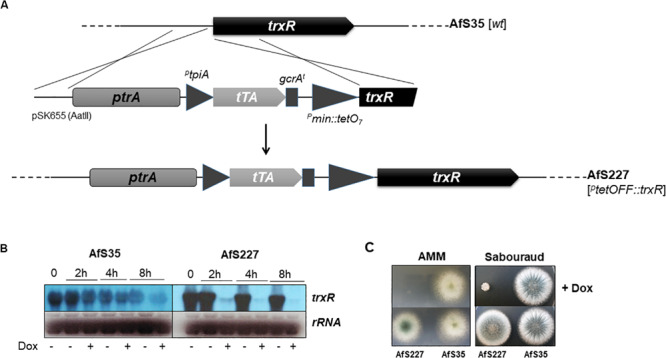
Generation of a conditional promoter replacement (CPR) strain to modulate *trxR* expression validates essentiality of the thioredoxin reductase-encoding gene for *A. fumigatus*. **(A)** Schematic outline of the CPR procedure employing the *tetOFF:trxR* module of pSK655. **(B)** Steady state *trxR* transcript levels in the *wild-type* strain AfS35 and its *tetOFF:trxR* derivative AfS227 illustrate a significant down-regulation of *trxR* transcription for the latter in the presence of doxycycline (+Dox). **(C)** Growth of AfS227 on minimal (left panel) and rich Sabouraud (right panel) culture medium is strongly inhibited by doxycycline, thereby validating essentiality of the *trxR* gene.

To conduct further analyses with respect to the cellular function of the *A. fumigatus* TrxR, a recombinant strain expressing the *trxR* gene in a conditional manner was constructed by replacing its endogenous promoter by a TetOFF module. The corresponding replacement cassette was synthesized by generating a derivative of the conditional expression module from plasmid pSK606 in which a part of the *trxR* coding region was fused to the tet operator-containing minimal promoter sequence and its 5′ untranslated region upstream to the *ptrA* resistance marker. The resulting fragment of plasmid pSK655 was transformend into AfS35 to replace the endogenous *trxR* locus (Figure 1A) and the genotype of the resulting strain AfS227 was confirmed by means of diagnostic PCR. Furthermore, conditional transcription of the *trxR* gene could be validated by Northern blot hybridization, which revealed increased transcript levels in the absence of doxycycline but only a faint signal upon exposure to this compound (Figure 1B). In agreement with this, growth of the TetOFF:TrxR strain AfS227 was completely blocked in the presence of doxycycline (Figure 1C), thereby confirming essentiality of the *trxR* gene in *A. fumigatus*. Inoculating the strains on the complex and rich Sabouraud culture medium further corroborated this conditional phenotype.

**FIGURE 2:**
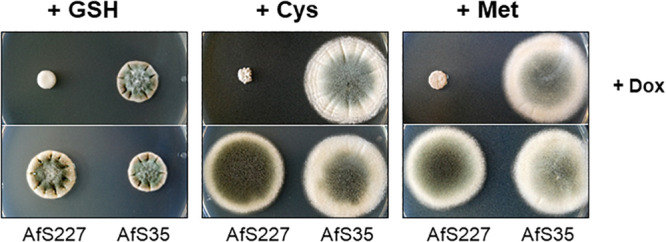
Growth impairment by TrxR deficiency is not rescued substantially by supplementation of glutathione or other organic sources of sulfur like cysteine or methionine. Shown are sporulating mycelia documented after three days of growth on culture medium supplemented with 20 mM glutathione (GSH) or 5 mM cysteine or methionine in the presence or absence of doxycycline.

Considering a link of the Trx/TrxR system to sulfur metabolism, we tested whether TrxR-deficiency of *A. fumigatus* might be rescued by organic sulfur sources or, as described for a thioredoxin-deficient strain of *A. nidulans* (Thön et al., 2007), by glutathione supplementation. Growth was monitored for the conditional promoter *tetOFF:trxR* strain and its wild-type progenitor on minimal culture medium supplemented with cysteine or methionine or reduced glutathione in the presence and absence of doxycycline (Figure 2). Interestingly, a severe retardation of radial hyphal extension became evident when *trxR* transcription was shut down, even in the presence of these sulfur-containing compounds. This observation is in contrast to the documented rescue of thioredoxin deficiency in *A. nidulans* by reduced glutathione (Thön et al., 2007) and furthermore indicates a cellular relevance of the Trx/TrxR system beyond sulfur assimilation.

### The *trxR* Gene Product Contributes to Oxidative Stress Resistance of *A. fumigatus*

**FIGURE 3:**
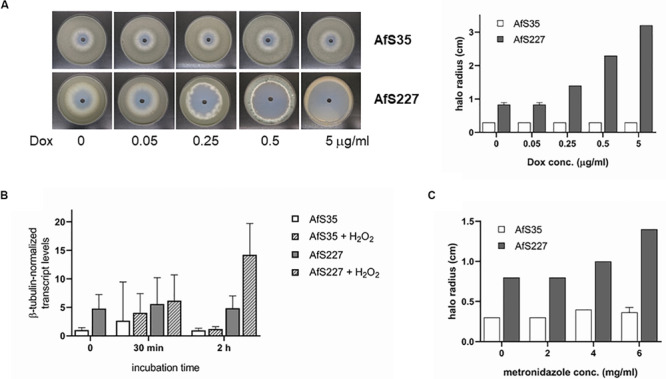
The *trxR* gene product contributes to oxidative stress resistance of *A. fumigatus*. **(A)** Growth inhibition by H_2_O_2_ correlates with increasing concentrations of doxycycline as monitored by halo formation in agar diffusion tests (left panel, plate assays; right panel, quantification from three replicates). **(B)** Transcription of *trxR* in the wild-type isolate AfS35 is not significantly influenced by oxidative stress conditions as triggered by 5 mM H_2_O_2_, whereas *trxR* transcript levels increase upon prolonged incubation of AfS227 in the presence of the oxidative stressor. All samples were generated from mycelia grown in the absence of doxycycline to exclude interference with *trxR* transcription in the TetOFF strain. **(C)** Oxidative stress resistance is influenced by metronidazole in a *trxR*-dependent manner, as deduced from a positive correlation of H_2_O_2_ sensitivity with increasing metronidazole concentrations for the conditional *tetOFF:trxR* expression strain AfS227.

An established cellular function of the thioredoxin system is to neutralize reactive oxygen species and therefore to contribute to oxidative stress resistance. To address this aspect, varying concentrations of doxycycline were used to dampen transcription of the *trxR* gene and resistance toward hydrogen peroxide was monitored by halo formation on solid culture plates. Whereas no influence of doxycycline on resistance of the progenitor strain AfS35 against H_2_O_2_ was seen, a clear positive correlation between doxycycline concentrations and sensitivity against hydrogen peroxide became evident (Figure 3A). Interestingly in the absence of doxycycline, oxidative stress resistance of the wild-type reference isolate was apparently higher than the one of the TetOFF strain, although steady state transcript levels had been monitored to be elevated for the latter. To monitor transcriptional regulation of the *trxR* gene under oxidative stress conditions which could account for this phenotypical behavior, transcript levels were quantified by quantitative real-time PCR (qRT-PCR) after shifting the strains from synthetic minimal medium to cultures lacking doxycycline but containing 5 mM H_2_O_2_ (Figure 3B). Analyses of samples taken after different time periods of incubation, 30 min and 2 h, did not reveal a significant variation in *trxR* transcript levels in AfS35 upon H_2_O_2_ exposure, while – for unknown reasons – *trxR* transcript levels increased significantly in the recombinant TetOFF strain AfS227 after 2 h. Thus, transcriptional upregulation of *trxR* was ruled out as underlying mechanism for the observed difference between AfS35 and the TetOff strain AfS227 in the presence of H_2_O_2_ only.

Metronidazole interferes with the cellular redox status, and TrxR of the parasite *E. histolytica* has been shown to form adducts with its metabolites (Leitsch et al., 2007). To assess the action of this nitroimidazole drug in *A. fumigatus*, the reference strain AfS35 and its *tetOFF:trxR* derivative AfS227 were exposed to varying concentrations and inhibition of growth was quantified in the presence of H_2_O_2_ in correlation to halo formation. No significant effect of metronidazole on oxidative stress resistance of AfS35 became evident, while the conditional promoter strain AfS227 displayed increasing sensitivity against H_2_O_2_ at higher metronidazole concentrations (Figure 3C). This phenotype underscores the requirement of a fine-tuned redox homeostasis that is supported by the thioredoxin system for resistance against oxidative stress conditions.

In a further step, we explored a possible interaction of TrxR inhibition by ebeselen and antifungal treatment as by the established first-line drug voriconazole. A corresponding broth microdilution checkerboard analysis covering concentration ranges from 1.0 to 0.016 μg/ml for voriconazole and 4.0 to 0.0625 μg/ml for ebselen revealed no synergistic interaction of these compounds: the minimal inhibiting concentrations (MICs) of voriconazole and ebselen against *A. fumigatus* strain AfS35 were 0.5 and 2.0 μg/ml, respectively, and these were not significantly altered when both compounds were present at varying concentrations in the culture medium.

### AfTrxR Is a Virulence Determinant of *A. fumigatus*

**FIGURE 4:**
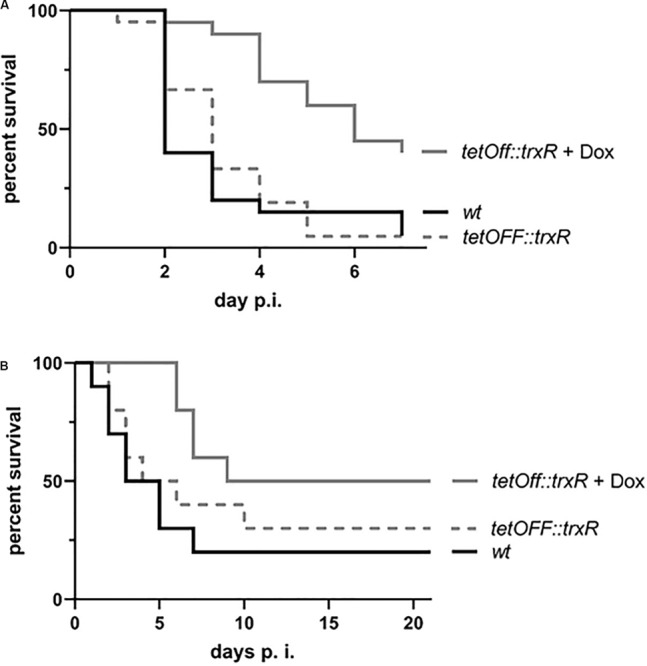
Pathogenicity of *A. fumigatus* is influenced by *trxR* transcription. **(A)** Infections of larvae of the greater wax moth *Galleria mellonella* (*n* = 20 for every cohort) with the CPR strain AfS227 reveal a highly significant (*p* = 0.0071) attenuation of virulence in the presence of doxycycline. **(B)** In a murine infection model of pulmonary invasive aspergillosis, animals fed with doxycycline show increased survival after infection with the conditional *tetOFF:trxR* expression strain (*n* = 10 for every cohort).

To address the vital question whether the *trxR*-encoded TrxR may serve as suitable target for antifungal therapy, we made use of our conditional promoter replacement strain in established models of infection. As preliminary surrogate hosts, larvae of the greater wax moth were infected with conidia of the respective strains, AfS35 and AfS227, as wild-type control and for conditional *trxR* expression, respectively. In addition, groups of larvae were treated with doxycycline at the day of infection and subsequently at every other day after infection. As expected, a significant proportion (95%) of the AfS35-treated larvae succumbed to the infection within seven days, irrespectively of the presence or absence of doxycycline (Figure 4A). The *tetOFF:trxR* strain mirrored virulence capacities of the wild-type progenitor in the absence of doxycycline, while doxycycline treatment resulted in a significantly elevated survival rate of 40% for the infected cohort. Correspondingly, the conditional knock down of *trxR* expression by doxycycline translates into a significantly reduced virulence of the recombinant TetOFF strain, supporting the idea that the fungal TrxR is required for the infectious process. Encouraged by these promising results, intranasal infections of susceptible, cortisone-acetate immunosuppressed mice were carried out to mimic the actual situation of invasive pulmonary aspergillosis (Figure 4B). In order to manipulate *trxR* transcription in the recombinant TetOFF strain AfS277, doxycycline was added to the drinking water of one cohort of AfS277-infected mice. The survival rate monitored for these animals was significantly increased in comparison to the wild-type strain AfS35 serving as control (50% vs. 20%, *p* = 0.0278), and a clear trend toward reduced virulence could be deduced when matching the course of infection to the non-treated cohort.

In essence, our study validates the postulated and demonstrated essentiality of the TrxR-encoding gene *trxR* in *A. fumigatus* and confirms for the first time that this trait translates into reduced virulence of this human pathogenic mold, making the thioredoxin system a valid target for antifungal therapy.

## Discussion

Fungal virulence is a multi-factorial trait, which is especially true for the opportunistic pathogen *A. fumigatus*, an environmental saprobe that appears to lack *bona fide* virulence factors (Tekaia and Latgé, 2005). Among general fitness determinants, such as osmotrophy, nutritional versatility, or thermotolerance, its pronounced resistance against a variety of stressors has to be regarded as determinant of virulence. In the context of host immunity, reactive oxygen species are generated by innate effector cells and numerous studies have demonstrated the importance of these agents for antifungal defense (Gallin and Zarember, 2007). Fungal-intrinsic, detoxifying activities such as catalases, peroxidases, or the glutathione system, however, counteract conditions of oxidative stress, and there appears to exist some degree of redundancy among the encoding genes (Paris et al., 2003). Yet, oxidative stress resistance has been a focus of attention with respect to establishing novel therapeutic targets. Such are urgently needed, considering the increasing incidence of fungal infections, the emergence of resistance, and the draining pharmaceutical pipeline of antifungal compounds. There have been several attempts to develop novel options of treatment accompanied by innovative delivery approaches, but given the apparent rise in fungal infections, a significant lack of knowledge, innovation, and investment becomes evident (Denning and Bromley, 2015; Osherov and Kontoyiannis, 2017).

Several approaches to identify and characterize target candidates in *A. fumigatus* have been pursued in the past, among them signature-tagged mutagenesis (Brown et al., 2000), gene replacement and conditional expression (GRACE) (Hu et al., 2007), *in silico* analyses (Thykaer et al., 2009; Abadio et al., 2011; Lu et al., 2014), or metabolic network modeling (Kaltdorf et al., 2016). Among these, one particular aspect of the fungal cell was identified repeatedly: redox homeostasis as conferred by the thioredoxin system. First evidence stems from a comprehensive candidate approach testing essential genes of *A. fumigatus* by conditional promoter replacement (Hu et al., 2007). There, one recombinant mutant could be recovered that displayed a strong static phenotype *in vitro* under repressive growth conditions. In a further study based on a metabolic flux model of *Aspergillus niger* and a systematic *in silico* deletion approach, essential genes of *A. fumigatus* have been postulated (Thykaer et al., 2009), among them the *trr1* gene, that is here denoted as *trxR*. This was in support of a recent study following a metabolic network strategy combined with orthology analysis and infection relevant transcriptome profiling data (Kaltdorf et al., 2016): Among the candidates resulting from the corresponding bioinformatic pipeline, the TrxR-encoding gene was retrieved.

The fact that the TrxR-encoding gene is, as demonstrated here, indeed essential for growth of *A. fumigatus* under *in vitro* conditions and shows only weak homology to its human ortholog, heightens its potential as an antifungal target. While there have been preliminary data about the essentiality of the *trxR* gene for *A. fumigatus* survival, a definitive proof-of-concept in models of infections has been lacking. Due to the essential nature of this gene, we made use of an effective conditional expression system based on doxycycline-dependent transcription of the gene of interest, driven by a TetOFF-responsive promoter (Wanka et al., 2016). While the alternative TetON version of this approach had been validated successfully before (Sasse et al., 2016), the conditional shut-down of transcription by the TetOFF module appears to be less strict under *in vivo* conditions, as deduced from a significant attenuation but not absence of virulence for the recombinant *tetOFF:trxR* strain in the presence of doxycycline. The insight that conditional repression of transcription varies with respect to the genes targeted and also depends on the regime of doxycycline administration became recently evident (Peng et al., 2018). In our studies, we refrained from optimizing the concentration or dosage of doxycycline, as the significant virulence attenuation in two alternative models of infection makes a strong case for the *trxR*-encoded TrxR of *A. fumigatus* to serve as an antifungal target. Supporting evidence might be generated by replacing the conditional TetOFF module by the metabolite-inducible *xylP* promoter as it has recently been validated *in vivo* (Bauer et al., 2019). Moreover, the conditional phenotypes displayed by the TetOFF strain underscore the importance of cellular redox homeostasis for fungal viability, becoming evident under permissive conditions that result in elevated *trxR* transcript levels.

Thioredoxin and its reductase were implied to be of relevance for fungal sulfur metabolism (Grant, 2001), while a corresponding *A. nidulans trxA*Δ deletion strain could be rescued by addition of glutathione to the growth medium (Thön et al., 2007). The observed phenotypes of our TetOFF strain under restrictive conditions might therefore be linked to limited sulfur supply. This consideration is of special interest given that a limited supply of sulfur-containing amino acids appears to be present in the course of pulmonary infection, while sulfate assimilation is not relevant for *A. fumigatus* virulence (Amich et al., 2016; Dietl et al., 2018). Yet, neither reduced glutathione nor cysteine or methionine supported growth of AfS227 to a significant extent and we therefore hypothesize that the phenotype from TrxR deficiency is rather linked to a more comprehensive cellular trait apart from sulfur supply and metabolism.

The pleiotrophic cellular functions that are associated with the thioredoxin system and apparently its reductase are likely to explain the severe phenotype resulting from shutting down *trxR* transcription that manifests in the absence of growth under standard culture conditions. The recombinant strain expressing the encoding gene in a conditional manner, however, allows to scrutinize specific phenotypes, such as altered oxidative stress resistance. There, our data revealing differences between the wild-type isolate AfS35 and the TetOFF strain AfS227 indicate a strong requirement for balanced TrxR expression for redox homeostasis.

Our study validates the *trxR* gene of *A. fumigatus* to be essential and makes a strong case for the encoded protein to serve as a cellular target for antifungal therapy. This is corroborated by the observation that TrxR activities are essential in other fungi as well (Missall and Lodge, 2005), suggesting that it is a pan-fungal candidate. While the number of distinct and specific inhibitors of TrxRs of fungal origin is limited, aspects of ligandability and druggability might be addressed for the *A. fumigatus* enzyme based on its recently solved X-ray structure (Marshall et al., 2019). In line with this, our study adds to the growing body of knowledge on fungal TrxRs and specifically supports the hypothesis that it represents a promising target for antifungal therapy.

## Materials and Methods

### Strains, Culture Media, and Growth Conditions

The *A. fumigatus* isolate AfS35, a non-homologous recombination-deficient derivative of the clinical isolate D141 (Reichard et al., 1990) served as reference recipient for the generation of recombinant fungal strains. Growth at 37°C was supported by minimal medium with 1% glucose as sole source of carbon and further supplements (0.52 g⋅l^–1^ KCl, 0.52 g⋅l^–1^ MgSO_4_, 1.52 g⋅l^–1^ KH_2_PO_4_, 0.1%trace element solution (Scott and Käfer, 1982), 5 mM ammonium tartrate or 10 mM sodium nitrate, pH 6.5), while selection for resistance conferred by the *ptrA* genetic marker (Kubodera et al., 2000) was carried out in the presence of 0.05 μg⋅ml^–1^ pyrithiamine.

### Generation of Recombinant Fungal Strains

Generating fungal protoplasts and their transformation were essentially performed as described previously (Dümig and Krappmann, 2015); *Escherichia coli* transformations were carried out using calcium/manganese-treated cells according to the protocol of Hanahan et al. (1991).

Cloning of gene replacement cassettes followed standard protocols of recombinant DNA technology (Sambrook et al., 1989). The *tetOFF:trxR* promoter replacement cassette of pSK655 was constructed by amplifying segments of the *trxR* 5′ and coding region by polymerase chain reactions using the oligonucleotide pairs JB169 (5′-AA TTCGAGCTCGGTACTTTAAAGGTTGGAAGAAATGGTTG G-3′) and JB170 (5′-GCCATCTAGGCCATCATCTGGAAGCAG AGGTTGAATTGAC-3′), and JB171 (5′-GCCTGAGTGGC CGTTTATGGTGCACACAAAAGTTGTCAG-3′) and JB172 (5′-GCCAAGCTTGCATGCCTTTAAACTTTTCGGCCTCGAGAG C-3′), respectively. The resulting fragments were assembled with the TetOFF module (Wanka et al., 2016) excised from pSK606 by *Pme*I and *Hin*dIII into the cloning vector pUC19L by seamless cloning, and from the resulting plasmid a 6.5 kb *Aat*II fragment could be released for transformation of the *A. fumigatus* recipient.

### Extraction of Nucleic Acids

*Aspergillus fumigatus* genomic DNA was prepared from ground mycelia as described (Wu et al., 1998) and plasmid DNA isolated from *E. coli* by employing the Machery-Nagel NucleoSpin plasmid DNA purification kit or the NucleoBond Xtra Midi plasmid DNA purification kit.

Small amounts of total RNA for qRT-PCR were extracted from ground mycelia by applying the innuPREP Plant RNA kit (Analytik Jena). Larger amounts of total RNA for Northern hybridization were extracted from ground mycelia with the TRI Reagent^®^ (Sigma-Aldrich) and cleaned with the peqGOLD Phase Trap Eppendorf tube (Peqlab). DNase I (Thermo Fisher Scientific) treatment was carried out according to the manufacturer’s instructions, followed by purification with the Qiagen RNeasy mini kit.

### Quantification of Transcript Levels

For Northern blot hybridizations, 10 μg of total RNA samples were separated in formaldehyde-containing agarose gels by electrophoresis, blotted onto Amersham Hybond-N nylon membranes (GE Healthcare), and hybridized with digoxigenin (DIG)-labeled probes. To monitor the transcript level of the *trxR* gene, the hybridizing probe was amplified from D141 genomic DNA with the primer pair JB064/065 (5′-TGTTCTCTATGAGGGTATGC-3′ and 5′-TTCGACTTGTATTCTGGAACG-3′) and labeled during amplification by applying the PCR DIG labeling mix (Roche).

Reverse transcription of DNase I-digested and purified RNA into cDNA was performed using the SuperScript III first-strand synthesis SuperMix for qRT-PCR (Invitrogen) according to the manufacturer’s protocol. Five hundred nanogram RNA template was used by default for each reaction in a total volume of 20 μl. qRT-PCR was applied for the quantification of transcript levels by the 7900HT fast real-time PCR system with a 384-well block module (Applied Biosystems) according to the manufacturer’s instructions with reactions performed in MicroAmp optical 384-well reaction plates (Applied Biosystems) with qPCR adhesive seal sheets (4titude). The standard volume of each individual reaction was 10 μl containing 10 ng cDNA as a template, primers at a concentration of 250 nM each, and 2 μl of the 5×EvaGreen qPCR mix (Rox, Bio&Sell). The primer pair used for quantification of *trxR* transcript levels under conditions of oxidative stress was JB302 and JB303 (5′-GACGCCAACGGTCTCTTCTA-3′ and 5′-GCTTTCGGCTTCGGCAATAAA-3′). All reactions were performed in technical triplicates, and dissociation curves were plotted to determine the specificity of PCR runs. For analyses of the qRT-PCR results, the SDS software (version 2.4, Applied Biosystems) was used and transcript levels were calculated following the comparative threshold cycle procedure (2^–ΔΔCT^ method, Livak and Schmittgen, 2001) with transcript levels of the β-tubulin-encoding *tubA* gene (AFUA_1G10910) serving as internal, constitutive reference.

### Models of Infection

Infections of larvae of the greater waxmoth *Galleria mellonella* (Mous Live Bait, Netherlands) were performed as described (Kavanagh and Reeves, 2004; Maerker et al., 2005). Larvae were injected with 1.5⋅10^6^ freshly harvested and washed conidia in a saline solution supplemented with 0.02% Tween 80 and 10 μl⋅ml^–1^ rifampicin to avoid bacterial infections and incubated at 30°C in the dark for up to 7 days.

Infection studies in a non-neutropenic murine model of pulmonary aspergillosis were essentially carried out as described (Bauer et al., 2019) in 6 week-old female ICR mice treated with subcutaneous injections of 300 mg/kg cortisone acetate 3 days prior to infection, at the day of infection, and every 3 days after. Infections were carried out by intranasal instillation of 2 × 10^5^ conidiospores suspended in 20 μl of saline plus 0.02% Tween 20. For some cohorts infected with the TetOFF:TrxR strain AfS227, doxycycline had been added to the drinking water at 0.2% before and throughout the period of infection.

Statistical analyses for comparison of survival curves among cohorts of infected animals were performed using the GraphPad Prism 6 software to be compared by the Log-rank (Mantel-Cox) test.

### Assessment of Growth and Minimal Inhibiting Concentrations

Fungal growth was monitored on solid culture plates point inoculated with 10^3^ conidia and incubated at 37°C for up to 4 days to follow radial extension of the hyphal mycelia. MICs for ebselen and voriconazole and combinations thereof were determined in a checkerboard approach following the EUCAST protocol by scoring conidial germination over three days in liquid RPMI cultures of a volume of 300 μl with inocula of 10^3^ conidia.

## Data Availability Statement

All datasets generated for this study are included in the article/supplementary material.

## Ethics Statement

The animal study was reviewed and approved by Ministry of Health (MOH) Animal Welfare Committee, Israel.

## Author Contributions

JB, YS, NO, and SK conceived and designed the experiments and analyzed the data. JB and YS performed the experiments. NO and SK wrote the manuscript. All authors contributed to the article and approved the submitted version.

## Conflict of Interest

The authors declare that the research was conducted in the absence of any commercial or financial relationships that could be construed as a potential conflict of interest.

## Footnotes

1 www.aspgd.org

## References

[B1] Abadio A. K. Kioshima E. S. Teixeira M. M. Martins N. F. Maigret B. Felipe M. S. (2011). Comparative genomics allowed the identification of drug targets against human fungal pathogens. *BMC Genomics* 12:75. 10.1186/1471-2164-12-75 21272313 PMC3042012

[B2] Amich J. Dümig M. O’Keefe G. Binder J. Doyle S. Beilhack A. (2016). Exploration of sulfur assimilation of *Aspergillus fumigatus* reveals biosynthesis of sulfur-containing amino acids as a virulence determinant. *Infect. Immun.* 84 917–929. 10.1128/iai.01124-15 26787716 PMC4807484

[B3] Arner E. S. Holmgren A. (2000). Physiological functions of thioredoxin and thioredoxin reductase. *Eur. J. Biochem.* 267 6102–6109. 10.1046/j.1432-1327.2000.01701.x 11012661

[B4] Azad G. K. Tomar R. S. (2014). Ebselen, a promising antioxidant drug: mechanisms of action and targets of biological pathways. *Mol. Biol. Rep.* 41 4865–4879. 10.1007/s11033-014-3417-x 24867080

[B5] Bauer I. Misslinger M. Shadkchan Y. Dietl A. M. Petzer V. Orasch T. (2019). The lysine deacetylase RpdA is essential for virulence in *Aspergillus fumigatus*. *Front. Microbiol.* 10:2773. 10.3389/fmicb.2019.02773 31866965 PMC6905131

[B6] Brown G. D. Denning D. W. Gow N. A. Levitz S. M. Netea M. G. White T. C. (2012). Hidden killers: human fungal infections. *Sci. Transl. Med.* 4:165rv113. 10.1126/scitranslmed.3004404 23253612

[B7] Brown J. S. Aufauvre-Brown A. Brown J. Jennings J. M. Arst H. Jr. Holden D. W. (2000). Signature-tagged and directed mutagenesis identify PABA synthetase as essential for *Aspergillus fumigatus* pathogenicity. *Mol. Microbiol.* 36 1371–1380. 10.1046/j.1365-2958.2000.01953.x 10931287

[B8] Brown N. A. Goldman G. H. (2016). The contribution of *Aspergillus fumigatus* stress responses to virulence and antifungal resistance. *J. Microbiol.* 54 243–253. 10.1007/s12275-016-5510-4 26920884

[B9] Brown S. M. Campbell L. T. Lodge J. K. (2007). *Cryptococcus neoformans*, a fungus under stress. *Curr. Opin. Microbiol.* 10 320–325. 10.1016/j.mib.2007.05.014 17707685 PMC2570326

[B10] Brunke S. Mogavero S. Kasper L. Hube B. (2016). Virulence factors in fungal pathogens of man. *Curr. Opin. Microbiol.* 32 89–95. 10.1016/j.mib.2016.05.010 27257746

[B11] Casadevall A. Steenbergen J. N. Nosanchuk J. D. (2003). ‘Ready made’ virulence and ‘dual use’ virulence factors in pathogenic environmental fungi – the *Cryptococcus neoformans* paradigm. *Curr. Opin. Microbiol.* 6 332–337. 10.1016/s1369-5274(03)00082-1 12941400

[B12] Dantas Ada S. Day A. Ikeh M. Kos I. Achan B. Quinn J. (2015). Oxidative stress responses in the human fungal pathogen, *Candida albicans*. *Biomolecules* 5 142–165. 10.3390/biom5010142 25723552 PMC4384116

[B13] Denning D. W. Bromley M. J. (2015). Infectious disease. How to bolster the antifungal pipeline. *Science* 347 1414–1416. 10.1126/science.aaa6097 25814567

[B14] Dietl A. M. Binder U. Shadkchan Y. Osherov N. Haas H. (2018). Siroheme is essential for assimilation of nitrate and sulfate as well as detoxification of nitric oxide but dispensable for murine virulence of *Aspergillus fumigatus*. *Front. Microbiol.* 9:2615. 10.3389/fmicb.2018.02615 30483221 PMC6240589

[B15] Dümig M. Krappmann S. (2015). “Controlling fungal gene expression using the doxycycline-dependent Tet-ON system in *Aspergillus fumigatus*,” in *Genetic Transformation Systems in Fungi*, Chapter 10 eds van den Berg M. Maruthachalam K. (New York, NY: Springer Publishers), 131–140.

[B16] Gallin J. I. Zarember K. (2007). Lessons about the pathogenesis and management of aspergillosis from studies in chronic granulomatous disease. *Trans. Am. Clin. Climatol. Assoc.* 118 175–185. 18528501 PMC1863604

[B17] Grant C. M. (2001). Role of the glutathione/glutaredoxin and thioredoxin systems in yeast growth and response to stress conditions. *Mol. Microbiol.* 39 533–541. 10.1046/j.1365-2958.2001.02283.x 11169096

[B18] Hanahan D. Jessee J. Bloom F. R. (1991). Plasmid transformation of *Escherichia coli* and other bacteria. *Methods Enzymol.* 204 63–113. 10.1016/0076-6879(91)04006-a 1943786

[B19] Hartmann T. Sasse C. Schedler A. Hasenberg M. Gunzer M. Krappmann S. (2011). Shaping the fungal adaptome – stress responses of *Aspergillus fumigatus*. *Int. J. Med. Microbiol.* 301 408–416. 10.1016/j.ijmm.2011.04.008 21565548

[B20] Hillmann F. Bagramyan K. Straßburger M. Heinekamp T. Hong T. B. Bzymek K. P. (2016). The crystal structure of peroxiredoxin Asp f3 provides mechanistic Insight into oxidative stress resistance and virulence of *Aspergillus fumigatus*. *Sci. Rep.* 6:33396. 10.1038/srep33396 PMC5022050 27624005

[B21] Hu W. Sillaots S. Lemieux S. Davison J. Kauffman S. Breton A. (2007). Essential gene identification and drug target prioritization in *Aspergillus fumigatus*. *PLoS Pathog.* 3:e24. 10.1371/journal.ppat.0030024 17352532 PMC1817658

[B22] Kaltdorf M. Srivastava M. Gupta S. K. Liang C. Binder J. Dietl A. M. (2016). Systematic identification of anti-fungal drug targets by a metabolic network approach. *Front. Mol. Biosci.* 3:22. 10.3389/fmolb.2016.00022 27379244 PMC4911368

[B23] Kavanagh K. Reeves E. P. (2004). Exploiting the potential of insects for in vivo pathogenicity testing of microbial pathogens. *FEMS Microbiol. Rev.* 28 101–112. 10.1016/j.femsre.2003.09.002 14975532

[B24] Köhler J. R. Hube B. Puccia R. Casadevall A. Perfect J. R. (2017). Fungi that infect humans. *Microbiol. Spectr.* 5:FUNK–0014–2016. 10.1128/microbiolspec.funk-0014-2016 PMC11687496 28597822

[B25] Kubodera T. Yamashita N. Nishimura A. (2000). Pyrithiamine resistance gene (*ptrA*) of *Aspergillus oryzae*: cloning, characterization and application as a dominant selectable marker for transformation. *Biosci. Biotechnol. Biochem.* 64 1416–1421. 10.1271/bbb.64.1416 10945258

[B26] Latgé J.-P. Chamilos G. (2019). *Aspergillus fumigatus* and Aspergillosis in 2019. *Clin. Microbiol. Rev.* 33:e00140-18. 10.1128/CMR.00140-18 PMC6860006 31722890

[B27] Leal S. M. Jr. Vareechon C. Cowden S. Cobb B. A. Latgé J. P. Momany M. (2012). Fungal antioxidant pathways promote survival against neutrophils during infection. *J. Clin. Invest.* 122 2482–2498. 10.1172/jci63239 22706306 PMC3534057

[B28] Leitsch D. Kolarich D. Wilson I. B. H. Altmann F. Duchene M. (2007). Nitroimidazole action in *Entamoeba histolytica*: a central role for thioredoxin reductase. *PLoS Biol.* 5:e211. 10.1371/journal.pbio.0050211 17676992 PMC1933457

[B29] Livak K. J. Schmittgen T. D. (2001). Analysis of relative gene expression data using real-time quantitative PCR and the 2^–Δ^ ^Δ^ ^*C*^_*T*_ Method. *Methods* 25 402–408. 10.1006/meth.2001.1262 11846609

[B30] Lu Y. Deng J. Rhodes J. C. Lu H. Lu L. J. (2014). Predicting essential genes for identifying potential drug targets in *Aspergillus fumigatus*. *Comput. Biol. Chem.* 50 29–40. 10.1016/j.compbiolchem.2014.01.011 24569026

[B31] Maerker C. Rohde M. Brakhage A. A. Brock M. (2005). Methylcitrate synthase from *Aspergillus fumigatus*. Propionyl-CoA affects polyketide synthesis, growth and morphology of conidia. *FEBS J.* 272 3615–3630. 10.1111/j.1742-4658.2005.04784.x 16008561

[B32] Marshall A. C. Kidd S. E. Lamont-Friedrich S. J. Arentz G. Hoffmann P. Coad B. R. (2019). Structure, mechanism, and inhibition of *Aspergillus fumigatus* thioredoxin reductase. *Antimicrob. Agents Chemother.* 63:e02281-18. 10.1128/AAC.02281-18 PMC6395915 30642940

[B33] McDonagh A. Fedorova N. D. Crabtree J. Yu Y. Kim S. Chen D. (2008). Sub-telomere directed gene expression during initiation of invasive aspergillosis. *PLoS Pathog.* 4:e1000154. 10.1371/journal.ppat.1000154 18787699 PMC2526178

[B34] Missall T. A. Lodge J. K. (2005). Thioredoxin reductase is essential for viability in the fungal pathogen *Cryptococcus neoformans*. *Eukaryot. Cell* 4 487–489. 10.1128/ec.4.2.487-489.2005 15701811 PMC549343

[B35] Mustacich D. Powis G. (2000). Thioredoxin reductase. *Biochem. J.* 346(Pt 1) 1–8. 10657232 PMC1220815

[B36] Nordberg J. Arner E. S. (2001). Reactive oxygen species, antioxidants, and the mammalian thioredoxin system. *Free Radic. Biol. Med.* 31 1287–1312. 11728801 10.1016/s0891-5849(01)00724-9

[B37] Osherov N. Kontoyiannis D. P. (2017). The anti-*Aspergillus* drug pipeline: is the glass half full or empty? *Med. Mycol.* 55 118–124. 27562862 10.1093/mmy/myw060

[B38] Osmani A. H. Oakley B. R. Osmani S. A. (2006). Identification and analysis of essential *Aspergillus nidulans* genes using the heterokaryon rescue technique. *Nat. Protoc.* 1 2517–2526. 10.1038/nprot.2006.406 17406500

[B39] Paris S. Wysong D. Debeaupuis J. P. Shibuya K. Philippe B. Diamond R. D. (2003). Catalases of *Aspergillus fumigatus*. *Infect. Immun.* 71 3551–3562. 10.1128/iai.71.6.3551-3562.2003 12761140 PMC155756

[B40] Peng Y. Zhang H. Xu M. Tan M. W. (2018). A Tet-Off gene expression system for validation of antifungal drug targets in a murine invasive pulmonary aspergillosis model. *Sci. Rep.* 8:443. 10.1038/s41598-017-18868-9 PMC5765126 29323188

[B41] Reichard U. Büttner S. Eiffert H. Staib F. Rüchel R. (1990). Purification and characterisation of an extracellular serine proteinase from *Aspergillus fumigatus* and its detection in tissue. *J. Med. Microbiol.* 33 243–251. 10.1099/00222615-33-4-243 2258912

[B42] Saccoccia F. Angelucci F. Boumis G. Carotti D. Desiato G. Miele A. E. (2014). Thioredoxin reductase and its inhibitors. *Curr. Protein Pept. Sci.* 15 621–646. 10.2174/1389203715666140530091910 24875642 PMC4275836

[B43] Schwenn J. D. Krone F. A. Husmann K. (1988). Yeast PAPS reductase: properties and requirements of the purified enzyme. *Arch. Microbiol.* 150 313–319. 10.1007/BF00408300 3060034

[B44] Sambrook J. Fritsch E. F. Maniatis T. (1989). *Molecular Cloning: a Laboratory Manual.* Cold Spring Harbor, NY: Cold Spring Harbor Laboratory Press.

[B45] Sasse A. Hamer S. N. Amich J. Binder J. Krappmann S. (2016). Mutant characterization and *in vivo* conditional repression identify aromatic amino acid biosynthesis to be essential for *Aspergillus fumigatus* virulence. *Virulence* 7 56–62. 10.1080/21505594.2015.1109766 26605426 PMC4871646

[B46] Scott B. R. Käfer E. (1982). “*Aspergillus nidulans*: an organism for detecting a range of genetic damage,” in *Chemical Mutagens*, eds Deserres F. J. Hollaender A. (New York, NY: Plenum).

[B47] Shlezinger N. Irmer H. Dhingra S. Beattie S. R. Cramer R. A. Braus G. H. (2017). Sterilizing immunity in the lung relies on targeting fungal apoptosis-like programmed cell death. *Science* 357 1037–1041. 10.1126/science.aan0365 28883073 PMC5628051

[B48] Tekaia F. Latgé J.-P. (2005). *Aspergillus fumigatus*: saprophyte or pathogen? *Curr. Opin. Microbiol.* 8 385–392. 10.1016/j.mib.2005.06.017 16019255

[B49] Thön M. Al-Abdallah Q. Hortschansky P. Brakhage A. A. (2007). The thioredoxin system of the filamentous fungus *Aspergillus nidulans*: impact on development and oxidative stress response. *J. Biol. Chem.* 282 27259–27269. 10.1074/jbc.m704298200 17631497

[B50] Thomas D. Barbey R. Surdin-Kerjan Y. (1990). Gene-enzyme relationship in the sulfate assimilation pathway of *Saccharomyces cerevisiae*. Study of the 3’-phosphoadenylylsulfate reductase structural gene. *J. Biol. Chem.* 265 15518–15524. 2203779

[B51] Thykaer J. Andersen M. R. Baker S. E. (2009). Essential pathway identification: from *in silico* analysis to potential antifungal targets in *Aspergillus fumigatus*. *Med. Mycol.* 47(Suppl. 1) S80–S87. 19253142 10.1080/13693780802455305

[B52] Wanka F. Cairns T. Boecker S. Berens C. Happel A. Zheng X. (2016). Tet-on, or Tet-off, that is the question: advanced conditional gene expression in *Aspergillus*. *Fungal Genet. Biol.* 89 72–83. 10.1016/j.fgb.2015.11.003 26555930

[B53] Wu Q. Sandrock T. M. Turgeon B. G. Yoder O. C. Wirsel S. G. Aist J. R. (1998). A fungal kinesin required for organelle motility, hyphal growth, and morphogenesis. *Mol. Biol. Cell* 9 89–101. 10.1091/mbc.9.1.89 9436993 PMC25223

